# Res-ECA-UNet++: an automatic segmentation model for ovarian tumor ultrasound images based on residual networks and channel attention mechanism

**DOI:** 10.3389/fmed.2025.1589356

**Published:** 2025-05-21

**Authors:** Shushan Wei, Zhaoting Hu, Lu Tan

**Affiliations:** ^1^Health Management Center, The Third Affiliated Hospital of Southern Medical University, Guangzhou, China; ^2^Department of Pharmacy, The Third Affiliated Hospital of Southern Medical University, Guangzhou, China

**Keywords:** UNet++, ovarian tumor, residual networks, attention mechanism, medical image segmentation

## Abstract

**Objective:**

Ultrasound imaging has emerged as the preferred imaging modality for ovarian tumor screening due to its non-invasive nature and real-time dynamic imaging capabilities. However, in many developing countries, ultrasound diagnosis remains dependent on specialist physicians, where the shortage of skilled professionals and the relatively low accuracy of manual diagnoses significantly constrain screening efficiency. Although deep learning has achieved remarkable progress in medical image segmentation in recent years, existing methods still face challenges in ovarian tumor ultrasound segmentation, including insufficient robustness, imprecise boundary delineation, and dependence on high-performance hardware facilities. This study proposes a deep learning-based automatic segmentation model, Res-ECA-UNet++, designed to enhance segmentation accuracy while alleviating the strain on limited healthcare resources.

**Methods:**

The Res-ECA-UNet++ model employs UNet++ as its fundamental architecture with ResNet34 serving as the backbone network. To effectively address the vanishing gradient problem in deep networks, residual modules are incorporated into the skip connections between the encoding and decoding processes. This integration enhances feature extraction efficiency while improving model stability and generalization capabilities. Furthermore, the ECA-Net channel attention mechanism is introduced during the downsampling phase. This mechanism adaptively emphasizes tumor region-related channel information through global feature recalibration, thereby improving recognition accuracy and localization precision for tumor areas.

**Results:**

Based on clinical ultrasound datasets of ovarian tumors, experimental results demonstrate that Res-ECA-UNet++ achieves outstanding performance in clinical validation, with a Dice coefficient of 95.63%, mean Intersection over Union (mIoU) of 91.84%, and accuracy of 99.75%. Compared to the baseline UNet, Res-ECA-UNet++ improves these three metrics by 0.45, 4.42, and 1.57%, respectively. Comparative analyses of ROC curves and AUC values further indicate that Res-ECA-UNet++ exhibits superior segmentation accuracy and enhanced generalization capabilities on the test set. In terms of computational efficiency, the inference time of Res-ECA-UNet++ meets clinical real-time requirements on both high-end and low-end hardware, demonstrating its suitability for deployment on resource-constrained devices. Additionally, comparative experiments on the public OTU2D dataset validate the model’s superior segmentation performance, highlighting its strong potential for practical applications.

**Conclusion:**

The proposed Res-ECA-UNet++ model demonstrates exceptional accuracy and robustness in the segmentation of ovarian tumor ultrasound images, highlighting its potential for clinical application. Its ability to enhance segmentation precision and aid clinicians in diagnosis underscores broad prospects for practical implementation. Future research will focus on optimizing the model architecture to further improve its adaptability to diverse pathological types and imaging characteristics, thereby expanding its clinical diagnostic utility.

## 1 Introduction

In recent years, ovarian cancer has demonstrated a persistent rise in both incidence and mortality rates, posing a significant threat to women’s health worldwide. With over 300,000 new cases of malignant ovarian tumors diagnosed annually, this disease has emerged as one of the most prevalent cancers affecting the female reproductive system globally (1). Marked disparities in ovarian cancer burden exist across nations of varying economic statuses. Low- and middle-income countries face disproportionate challenges due to constrained healthcare resources, resulting in elevated disease burden and suboptimal clinical outcomes (2). China mirrors this global pattern, where ovarian cancer incidence rates have exhibited a consistent upward trajectory in recent years (3). Due to the lack of specific symptoms and effective biomarkers in early-stage ovarian cancer, approximately 70–80% of patients are diagnosed at an advanced stage, leading to a sharp decline in the five-year survival rate to 30–45% (4, 5). Studies indicate that early screening is a critical measure to reduce ovarian cancer mortality, as early diagnosis can elevate the five-year survival rate to over 90% (6, 7). Ultrasonic examination has become the preferred imaging modality for ovarian tumor screening due to its non-invasive nature, real-time imaging capabilities, and cost-effectiveness (8).

However, traditional ultrasound diagnosis heavily relies on clinicians’ experience and is susceptible to subjective factors, leading to relatively low diagnostic consistency. In regions with limited medical resources, the shortage of specialized physicians further exacerbates the risks of missed diagnoses and misdiagnoses (9, 10). Although ultrasonography plays a significant role in ovarian tumor screening, these challenges have prompted the adoption of novel technological solutions. In this context, deep learning-based intelligent segmentation techniques for ovarian tumor ultrasound imaging have emerged (11). Compared with conventional ultrasound diagnostic methods, this technology demonstrates enhanced capabilities in improving diagnostic accuracy and reducing human errors. Furthermore, it provides technical support for establishing standardized screening protocols, thereby facilitating broader clinical implementation of ovarian tumor diagnosis.

## 2 Related work

Medical image segmentation techniques can be primarily categorized into traditional machine learning methods and deep learning approaches.

### 2.1 Traditional machine learning methods

Traditional machine learning algorithms mainly utilize image processing techniques such as morphological operations and threshold segmentation to identify specific regions and edge features (12). For instance, Wu et al. (13) developed an SVM classifier based on improved morphological features to achieve precise segmentation of breast tumor ultrasound images, achieving an accuracy of 95.24%. Poudel et al. (14) significantly enhanced thyroid ultrasound image segmentation accuracy through dynamic contour adjustment to optimize the segmentation framework. Zhu et al. (15) implemented accurate segmentation and recognition of hepatic cysts in ultrasound images using threshold algorithms. Gopalakrishnan et al. (16) employed multi-threshold methods for polycystic ovary syndrome ultrasound image segmentation, achieving outstanding results.

Although traditional methods perform well in specific tasks, their heavy reliance on manual feature extraction limits generalization capabilities and adaptability to complex variations in medical imaging. Additionally, these methods lack self-adaptive learning capacity, demonstrating significant limitations when handling ultrasound image artifacts such as noise and acoustic shadows.

### 2.2 Deep learning approaches

In contrast, deep learning approaches, particularly convolutional neural network (CNN)-based medical image segmentation techniques, exhibit superior adaptability and automatic feature learning capabilities. Unlike traditional methods, deep learning employs end-to-end training that avoids subjective bias in manual feature extraction while demonstrating strong processing capabilities for large-scale data (17–20). For example, Ma et al. (21) applied CNNs to thyroid nodule ultrasound image segmentation, with comparative experiments confirming their significant performance advantages over traditional machine learning methods. In recent years, researchers have proposed various innovative models based on CNNs. U-Net, as a classic architecture for medical image segmentation, demonstrates exceptional generalization capabilities in small-sample medical data tasks through its encoder-decoder structure and skip connections. By extracting multi-scale features through the encoder, recovering spatial information via the decoder, and compensating for information loss through skip connections, U-Net has been widely adopted in medical image analysis (22–24).

### 2.3 Challenges and advances in ovarian tumor ultrasound image segmentation

In the field of ovarian tumor ultrasound image segmentation, deep learning methods remain in the exploratory stage. In recent years, numerous studies have proposed diverse architectures and methodologies to enhance segmentation accuracy. For instance, Ta et al. (25) introduced a Weighted Fusion Architecture that integrates multiple network frameworks within the U-Net structure, effectively improving segmentation precision, albeit at the cost of increased computational complexity. To address small-sized tumors, Luong et al. (26) developed SovaSeg-Net, which combines VGG16 and Spatial Pyramid Pooling Fusion (SPPF) modules. While this approach enhances tumor segmentation capabilities, it demands substantial training data and computational resources. In contrast, Siahpoosh et al. (27) innovatively fused the ConvMixer with a Pyramid Dilated Convolution (PDC) module, demonstrating superior performance in multi-scale feature extraction and global contextual information capture.

Beyond architectural innovations, studies have also focused on model optimization. Nguyen et al. (28) optimized the traditional Segment Anything Model (SAM) by incorporating IoU, SSIM, and Focal Loss metrics, along with dual prompting strategies to guide model attention toward target regions. This method proves particularly effective for irregularly shaped tumors or low-quality images. Additionally, Shantharam et al. (29) utilized the UNet++ architecture, achieving higher segmentation accuracy through enhanced skip connections and hyperparameter tuning to optimize real-world performance.

Recent advancements in multimodal deep learning models have introduced novel perspectives for ultrasound image segmentation. For example, Wang et al. (30) proposed a framework combining ultrasound images, menopausal status, and serum tumor markers. Although this multimodal approach outperforms single- and dual-modal models in accuracy, its complexity and high computational requirements pose challenges for grassroots hospitals, particularly those in remote regions with limited infrastructure and technical support.

Despite recent progress in deep learning for medical image segmentation, numerous challenges persist in clinical applications. These include but are not limited to: the high morphological complexity of malignant ovarian tumors and inherent ultrasound imaging limitations such as speckle noise, acoustic shadows, and fuzzy boundaries that interfere with segmentation accuracy (31–33). Furthermore, resource-constrained settings often lack adequate hardware and specialized personnel, hindering the accessibility of deep learning technologies. Current segmentation methods have yet to fully resolve these challenges. Therefore, realizing widespread clinical application requires further research and technological innovation to provide more precise technical support for ultrasound-assisted diagnosis.

## 3 Methods

### 3.1 UNet++ network

The traditional UNet model employs a direct concatenation strategy for feature fusion between encoder and decoder feature maps during its architecture design. While straightforward to implement, this approach is prone to boundary ambiguity in segmentation results, limiting its generalization capability in complex medical imaging tasks (34). Additionally, UNet exhibits depth sensitivity, requiring time-consuming manual hyperparameter tuning for different datasets, which increases computational costs. These limitations hinder UNet’s performance in multi-scale medical image segmentation. To address these challenges, researchers proposed UNet++, which optimizes feature fusion strategies and enhances generalization through dense skip connections and a deep supervision mechanism. Similar to UNet, UNet++ retains a U-shaped structure comprising an encoder, decoder, and skip connections (35). In the encoder, UNet++ performs downsampling via convolutional layers combined with ReLU activation functions and max-pooling operations. This process reduces spatial dimensions while increasing channel depth to capture richer semantic information. In the decoder, upsampling operations restore image resolution, localize features, and recover fine-grained details to generate the final output.

Compared to UNet’s single-layer skip connections, UNet++ adopts a dense skip connection strategy that establishes richer interconnections between feature layers at different scales. By linking multiple intermediate decoder nodes, the decoder can not only utilize feature information from the preceding layer but also directly access high-resolution features from lower-level encoders. This architecture effectively reduces the semantic gap between encoding and decoding stages, addressing UNet’s insufficient information fusion caused by direct encoder-decoder connections. The proposed mechanism not only enhances the capability of multi-scale feature fusion but also better preserves structural details and contextual semantic information, which plays a critical role in segmenting ovarian tumor ultrasound images characterized by ambiguous boundaries and complex morphological configurations.

Furthermore, UNet++ introduces deep supervision across multiple decoder levels, in contrast to UNet which only implements supervisory learning at the final output layer. This mechanism enhances network performance through two principal aspects: First, the incorporation of auxiliary loss terms at multi-scale decoding layers facilitates more efficient gradient propagation to shallow network layers, significantly accelerating model convergence while improving generalization capability. Second, the deep supervision mechanism compels the model to learn discriminative features across different spatial scales, thereby strengthening its adaptability to multi-scale anatomical structures. These synergistic advantages make UNet++ particularly effective in segmenting low-contrast, noise-prone ultrasound images where traditional architectures often struggle with boundary ambiguity and texture complexity.

However, despite its advanced feature fusion capabilities, UNet++ retains certain limitations. Partial detail loss may still occur during skip connections and upsampling, impacting segmentation precision (36, 37). Consequently, further optimizations, particularly those addressing boundary ambiguity and low-contrast tumor regions, remain necessary to enhance its adaptability.

### 3.2 Residual networks

The evolution of CNN architectures has driven advancements in computer vision and image recognition. As a classic deep CNN, VGGNet enhances model representation capabilities by increasing the number of convolutional layers (38). However, merely expanding network depth does not indefinitely improve learning capacity. As depth increases, issues such as vanishing gradients and exploding gradients become more pronounced, leading to degraded overall model performance. To address these challenges, GoogleNet introduced the Inception module, which extracts multi-scale features by utilizing parallel convolutional kernels and pooling operations of varying scales across different receptive fields (39). While this strategy expands network depth and width to improve performance, it does not fundamentally resolve the training difficulties inherent to deep networks.

**Figure 1:**
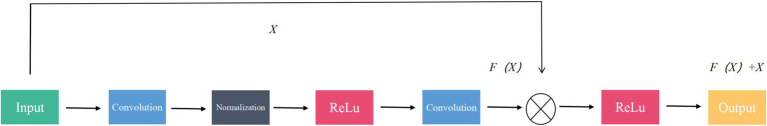
Diagram of the ResNet residual architecture.

To overcome these limitations, He et al. (40) proposed Residual Network (ResNet) and introduced the residual block structure (as illustrated in Figure 1). The core innovation of residual blocks lies in their use of shortcut connections to directly propagate feature information, enabling the network to learn residual mappings rather than directly fitting target outputs. This design mitigates vanishing gradient issues in deep networks while enhancing trainability and convergence speed. In medical image segmentation tasks, UNet++ integrates ResNet-inspired residual blocks to enable deeper extraction of critical features from complex images, thereby improving segmentation accuracy. Furthermore, residual connections help preserve low-level spatial information during deep feature extraction, enhancing the robustness of segmentation models.

### 3.3 Attention mechanism

**Figure 2:**
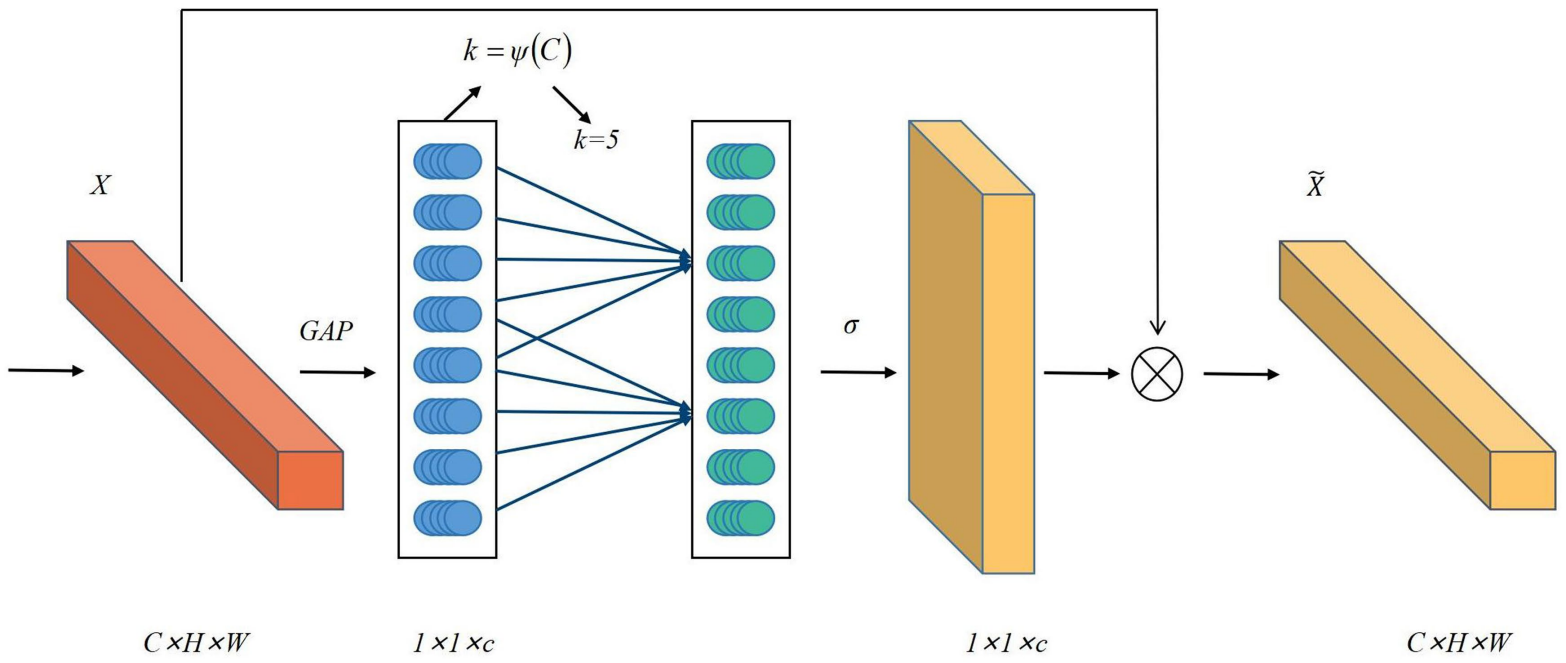
Diagram of the ECA-Net attention mechanism.

In ultrasound image segmentation, complex backgrounds and noise often hinder accurate feature extraction, compromising segmentation performance. To mitigate this, attention mechanisms, inspired by the human visual system’s selective processing, adaptively highlight salient features while suppressing irrelevant details. These mechanisms compute feature correlations to assign context-aware weights, proving especially effective for ultrasound images with ambiguous boundaries and low contrast. While methods like SE-Net (41) and CBAM (42) have been widely used in medical imaging, their reliance on fully connected (FC) layers introduces computational burdens and feature degradation risks. To address these limitations, ECA-Net emerges as a lightweight alternative that employs 1D convolution for local cross-channel interactions while eliminating dimensionality reduction (43). Its three-stage workflow comprises global feature aggregation through average pooling in the Squeeze phase, adaptive channel recalibration using 1D convolution during Excitation, and weighted feature fusion via sigmoid activation. This architecture achieves efficient attention computation with minimal parameter overhead. Compared to SE-Net and CBAM, ECA-Net preserves full channel representations while reducing computational costs, making it ideal for resource-constrained medical imaging applications (44, 45). The architecture and mathematical formulation of ECA-Net are detailed in Figure 2 and Equation 1.


(1) $$k=\psi (C)={|\frac{\log_{2}(C)}{\gamma }+\frac{b}{\gamma }|}_{\mathrm{odd}}$$

Where W is the width of the feature map, H is the height of the feature map, C is the number of channels, γ=2 , b=1 , and the calculation formula for C is: $C=2^{\left(\gamma *k-b\right)}$ , k represents the size of the one-dimensional convolution kernel and the frequency of local cross-channel interaction. U is the original feature and $\tilde{U}$ is the weighted feature. GAP represents the global average pooling operation.

To further validate the advantages of ECA-Net in ultrasound image segmentation tasks, this study conducts comparative experiments using SE-Net, CBAM, and ECA-Net as channel attention modules under identical network backbone architectures and experimental configurations.

**Figure 3:**
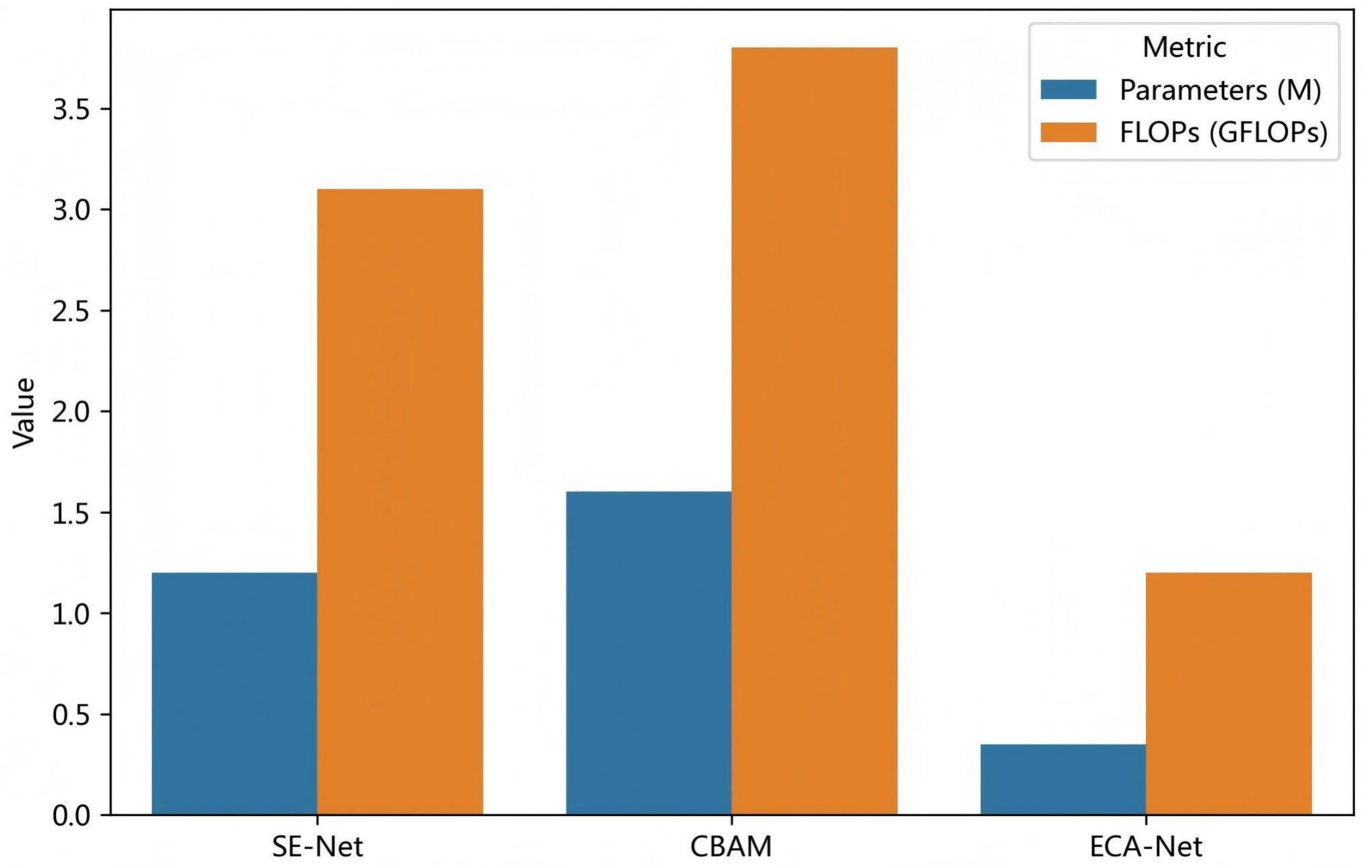
Comparison of parameters and computational cost among different attention mechanisms.

As illustrated in Figure 3, ECA-Net demonstrates significant superiority in both model parameters and computational complexity. The parameter size of ECA-Net approximates 0.35 million, markedly lower than CBAM’s 1.6 million and SE-Net’s 3.5 million. Computational costs decrease from 3.8 GFLOPs for CBAM to 1.2 GFLOPs for ECA-Net, representing a nearly 70% reduction. This structural efficiency eliminates the requirement for additional spatial attention branches while maintaining lightweight characteristics. Such technical advantages position ECA-Net as a more practical solution for deployment in clinical environments with constrained computational resources.

### 3.4 Improved model

**Figure 4:**
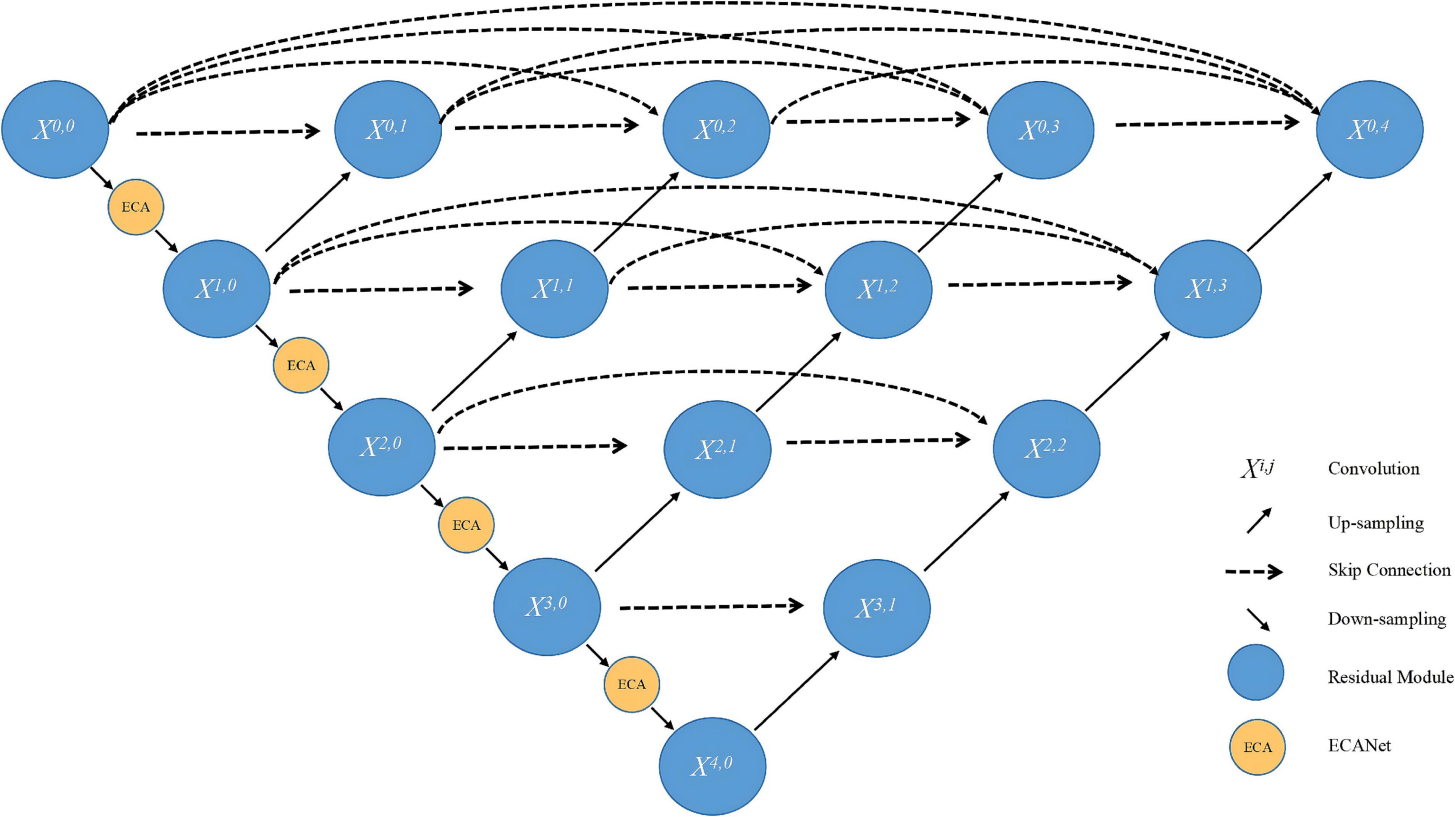
Network architecture of Res-ECA-UNet++.

To further enhance the performance of UNet++ in segmentation tasks, we propose an improved model named Res-ECA-UNet++ that integrates ResNet and ECA-Net attention mechanisms, as illustrated in Figure 4. The proposed architecture employs ResNet34 as its backbone network, which incorporates convolutional layers and residual blocks. By leveraging skip connections to directly propagate features, the model alleviates gradient vanishing issues, strengthens feature extraction capabilities, and improves training stability.

In ResNet34 implementation, we design a 34-layer convolutional architecture with four residual blocks. Each residual block incorporates Batch Normalization layers to stabilize training. Notably, the first convolutional kernel size is adjusted from 7 × 7 to 3 × 3 to enhance the model’s capacity for capturing small target features. These design improvements enhance feature representation capabilities, providing richer foundational features for subsequent ECA-Net modules and ultimately improving segmentation performance.

To strengthen the model’s ability to extract critical information, we integrate ECA-Net modules into the encoder path’s main convolutional layers $X^{i,0}$ , positioned after each downsampling residual module. This module dynamically allocates channel-wise weights during feature compression, amplifying responses to tumor-related features while suppressing background noise and artifact interference.

Our design rationale for ECA integration in the encoder path considers three key factors: (1) The encoder stage handles semantic abstraction and global feature perception, serving as the core component for generating critical features. Introducing channel attention mechanisms at this stage enables early-stage focus on lesion-related high-response channels, particularly effective for addressing common ultrasound image challenges such as blurred boundaries and weak grayscale variations. (2) The decoder’s primary task involves spatial information recovery with emphasis on detail reconstruction and upsampling concatenation. Applying channel attention at this stage could introduce redundant computation and weaken high-resolution information from skip connections. (3) To prevent over-compression or information suppression, we preserve the original decoder structure.

While ResNet34-extracted features may contain mixed tumor-background noise, ECA-Net significantly enhances tumor-related feature responses. This enables dynamic focus on critical regions while effectively suppressing noise and irrelevant background interference, thereby providing higher-quality inputs to the decoder. This deep synergistic interaction strengthens both feature extraction and selective attention capabilities, ensuring stable processing of ovarian tumor ultrasound images with blurred boundaries and complex morphologies, ultimately achieving remarkable segmentation accuracy improvement.

In the network, i in node $X^{i,j}$ represents the number of downsampled layers of the encoder and j represents the number of convolutional layers in the skip connection, as defined in Equation 2:


(2) $$\begin{array}{l} X^{i,j}=\{\begin{array}{l} H(X^{i-1,j}),j=0\\ H([{\left[X^{i,k}\right]}_{k=0}^{j=1}],u(X^{i+1,j-1})),j>0 \end{array} \end{array}$$

The conditional structure of this architecture reflects the dynamic nature of node input information. Instead of using simple activation functions, H(⋅) employs submodules comprising convolution, normalization, and nonlinear activation. This module enhances the model’s representational capacity. u(⋅) is the upsampling layer, and [⋅] is the linking operation. When node j=0 , the node receives input exclusively from the encoder of the previous layer. When node j>0 , the node integrates multiple inputs including both features from the previous encoder layer and information from all preceding nodes in the path, establishing dense skip connections.

This design achieves effective integration of multi-scale features, mitigates semantic loss caused by downsampling, and enhances the model’s sensitivity to ambiguous boundaries in medical images. Such architecture proves particularly advantageous for precise segmentation of fine-grained targets like ovarian tumors, demonstrating superior performance in handling complex anatomical structures.

## 4 Experimental results and analysis

### 4.1 Experimental environment

The experiments were conducted on a 64-bit Linux 20.04 server to ensure result stability and reproducibility. Model training and testing were implemented using the PyTorch 2.0.0 deep learning framework within the PyCharm development environment. The hardware platforms used for the experiments included a high-end system with an RTX 3090 GPU (24GB VRAM), 64GB RAM, and an Intel i7-10700 8-core 16-thread processor, as well as a low-end system with an NVIDIA Jetson Nano for comparison. The maximum number of training iterations was set to 200 epochs to ensure sufficient convergence. To prevent overfitting, an early stopping mechanism was implemented: training would be automatically terminated if no significant improvement in the Dice coefficient on the validation set was observed over 20 consecutive epochs. Hyperparameter configurations were determined based on preliminary experimental results and empirical knowledge. Considering the balance between training stability and GPU memory utilization, the batch size was set to 8. The initial learning rate was established at 0.001, employing a combined optimization strategy of AdamW optimizer with cosine annealing learning rate scheduling to enhance convergence speed and prevent entrapment in local optima. All hyperparameters underwent rigorous testing and fine-tuning on a dedicated validation subset to ensure optimal generalization capability and segmentation accuracy for ovarian tumor ultrasound image analysis tasks. Experimental results were visualized using Matplotlib.

### 4.2 Dataset description and processing

This study constructed an ovarian tumor ultrasound image dataset derived from patient imaging archives at the Third Affiliated Hospital of Southern Medical University between 2020 and 2023, comprising 350 confirmed malignant ovarian tumor ultrasound images. The distribution of tumor categories in the dataset is presented in Table 1. The dataset comprises ultrasound images of ovarian tumors characterized by the following features: small object recognition challenges, irregular morphological presentations, ambiguous boundary delineation, and difficulty in segmenting low-contrast regions. The use of this dataset was approved by the hospital’s Ethics Committee and strictly adhered to privacy protection protocols.

**Table 1:** Category distribution in the ovarian tumor dataset.

Tumor type	Number	Proportion (%)
Serous carcinoma	219	62.57%
Mucinous carcinoma	21	6.00%
Borderline serous tumor	23	6.57%
Borderline mucinous tumor	16	4.57%
Adult granulosa cell tumor	15	4.29%
Immature teratoma	14	4.00%
Clear cell carcinoma	9	2.57%
Endometrioid carcinoma	8	2.29%
Malignant transformation of mature teratoma	8	2.29%
Mesonephric adenocarcinoma	6	1.71%
Mixed germ cell tumor	6	1.71%
Yolk sac tumor	5	1.43%
Total	350	100.00%

**Figure 5:**
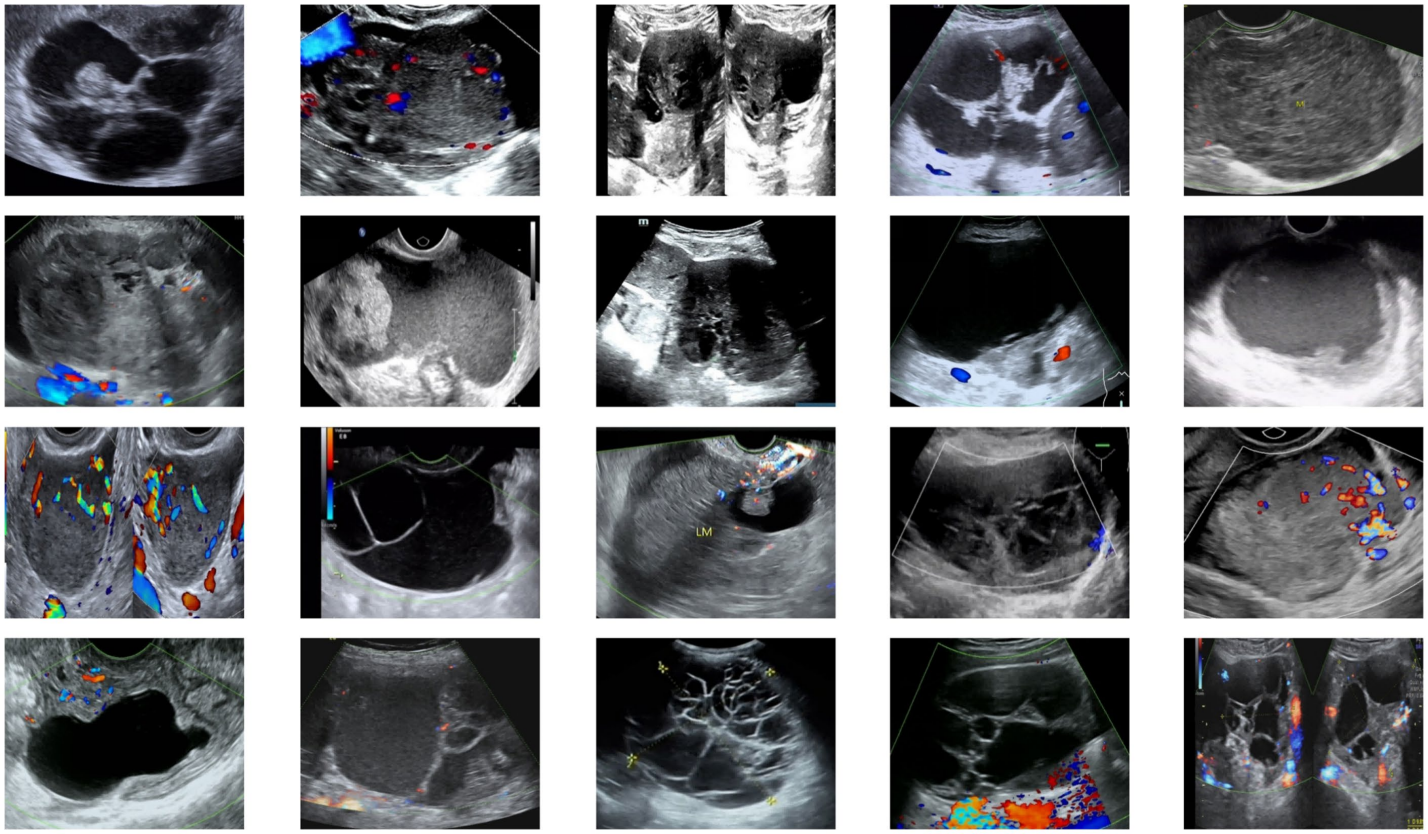
Examples from the ovarian tumor ultrasound dataset.

All images were annotated and verified by two professional physicians, with labels including tumor size, morphology, boundaries, and precise locations to ensure annotation quality and consistency. Annotated images were paired with corresponding label files and resized to a uniform input dimension of (512, 512, 3). To address data scarcity, augmentation techniques including horizontal flipping, affine transformation, and contrast enhancement were applied, expanding the dataset to 2,000 images. The data were partitioned into training and test sets at an 8:2 ratio to ensure generalization capability. Example images from the dataset are shown in Figure 5.

### 4.3 Evaluation metrics

To comprehensively evaluate the segmentation performance of the model, we adopt the Dice Similarity Coefficient (Dice), mean Intersection-over-Union (mIoU), Accuracy and Hausdorff distance as evaluation metrics. Among these, Dice and mIoU primarily measure the alignment between segmented regions and ground truth target areas, serving as core metrics for evaluating prediction accuracy in tumor region delineation. These metrics remain unaffected by background noise interference. Accuracy reflects the overall correctness of pixel-level classification. To further assess the model’s classification capabilities, Sensitivity and Specificity were employed to quantify its performance in correctly identifying tumor regions and accurately excluding non-tumor areas, respectively.

The Dice coefficient is used to assess the similarity between the predicted results and the ground truth segmentation. Its value ranges from [0, 1], where a value closer to 1 indicates better segmentation performance. The formula is as follows (Equation 3):


(3) $$\text{Dice}=\frac{2|P\cap G|}{|P|+|G|}$$

P represents the segmentation region predicted by the model, and G denotes the ground truth segmentation region. The notation ∣P∩G∣ indicates the number of pixels in the intersection area between the two regions. ∣P∣+∣G∣ represent the number of pixels in the predicted and ground truth labels, respectively.

mIoU quantifies the overlapping degree between predicted regions and ground truth regions, calculated as the average of Intersection over Union (IoU) values across multiple classes. The formula is expressed as follows (Equation 4):


(4) $$\text{mIoU}=\frac{1}{k+1}\sum_{i=0}^{k}\frac{p_{\mathrm{ii}}}{\sum_{j=0}^{k}p_{\mathrm{ij}}+\sum_{j=0}^{k}p_{\mathrm{ji}}-p_{\mathrm{ii}}}$$

The term $p_{\mathrm{ij}}$ denotes the number of true values for i that are predicted to be j , while k+1 represents the number of categories. The term $p_{\mathrm{ii}}$ denotes the number of true examples, and $p_{\mathrm{ij}}$ and $p_{\mathrm{ji}}$ represent the False Positive and False Negative, respectively.

Accuracy is defined as the ratio of correctly predicted pixels to the total number of pixels in the image. Sensitivity represents the proportion of tumor pixels correctly identified by the model relative to the actual total number of tumor pixels. Specificity indicates the proportion of non-tumor pixels appropriately excluded by the model compared to all non-tumor pixels present. The formulas are as follows, as defined in Equations 5, 6, and 7:


(5) $$\text{Accuracy}=\frac{T_{p}+T_{N}}{T_{P}+T_{N}+F_{P}+F_{N}}\times 100\%$$

(6) $$\text{Sensitivity}=\frac{T_{p}}{T_{P}+F_{N}}$$

(7) $$\text{Specificity}=\frac{T_{N}}{T_{N}+F_{P}}$$

Where $T_{p}$ represents the True Positive, $F_{N}$ represents the False Negative, $F_{P}$ represents the False Positive, and $T_{N}$ represents the True Negative.

The Hausdorff distance is a widely used metric for measuring similarity or distance between two sets. In image segmentation tasks, it quantifies the maximum deviation between predicted segmentation boundaries and ground truth boundaries. Given two point sets A and B , the Hausdorff distance is defined as (Equation 8):


(8) $$H(A,B)=\max(\underset{a\in A}{\sup}\underset{b\in B}{\inf d}(a,b),\underset{b\in B}{\sup}\underset{a\in A}{\inf}d(a,b))$$

Where sup denotes the supremum, inf represents the infimum, d(a,b) is the Euclidean distance between points a and b .

### 4.4 Experimental results

#### 4.4.1 Model performance comparison

To validate the segmentation performance of Res-ECA-UNet++, we conducted a comparative analysis with UNet, UNet++, and Res-UNet++, evaluating model improvements across three key metrics: Dice coefficient, mIoU, and accuracy (as shown in Table 2). Experimental results demonstrate that Res-ECA-UNet++ achieves superior performance compared to other models in terms of global segmentation accuracy, boundary recognition capability, and computational stability. Specifically, Res-ECA-UNet++ outperforms UNet, UNet++, and Res-UNet++ by 0.45, 0.17, and 0.11% in accuracy, 4.42, 1.44, and 1.29% in mIoU, and 1.57, 0.83, and 0.73% in Dice coefficient, respectively. These results indicate that the integration of residual modules and attention mechanisms in Res-ECA-UNet++ effectively enhances the model’s focus on critical tumor regions, optimizes segmentation boundary precision, and significantly improves overall segmentation performance.

**Table 2:** Performance comparison of different models on ovarian tumor ultrasound image segmentation.

Model	Accuracy (%)	mIoU (%)	Dice (%)	Hausdorff distance (pixels)	Sensitivity (%)	Specificity (%)
UNet	99.30	87.42	93.06	12.42	92.11	97.82
UNet++	99.58	90.40	94.80	10.22	93.62	98.41
Res-UNet++	99.64	90.55	94.90	9.10	94.04	98.63
Res-ECA-UNet++	99.75	91.84	95.63	7.96	95.23	98.92

**Figure 6:**
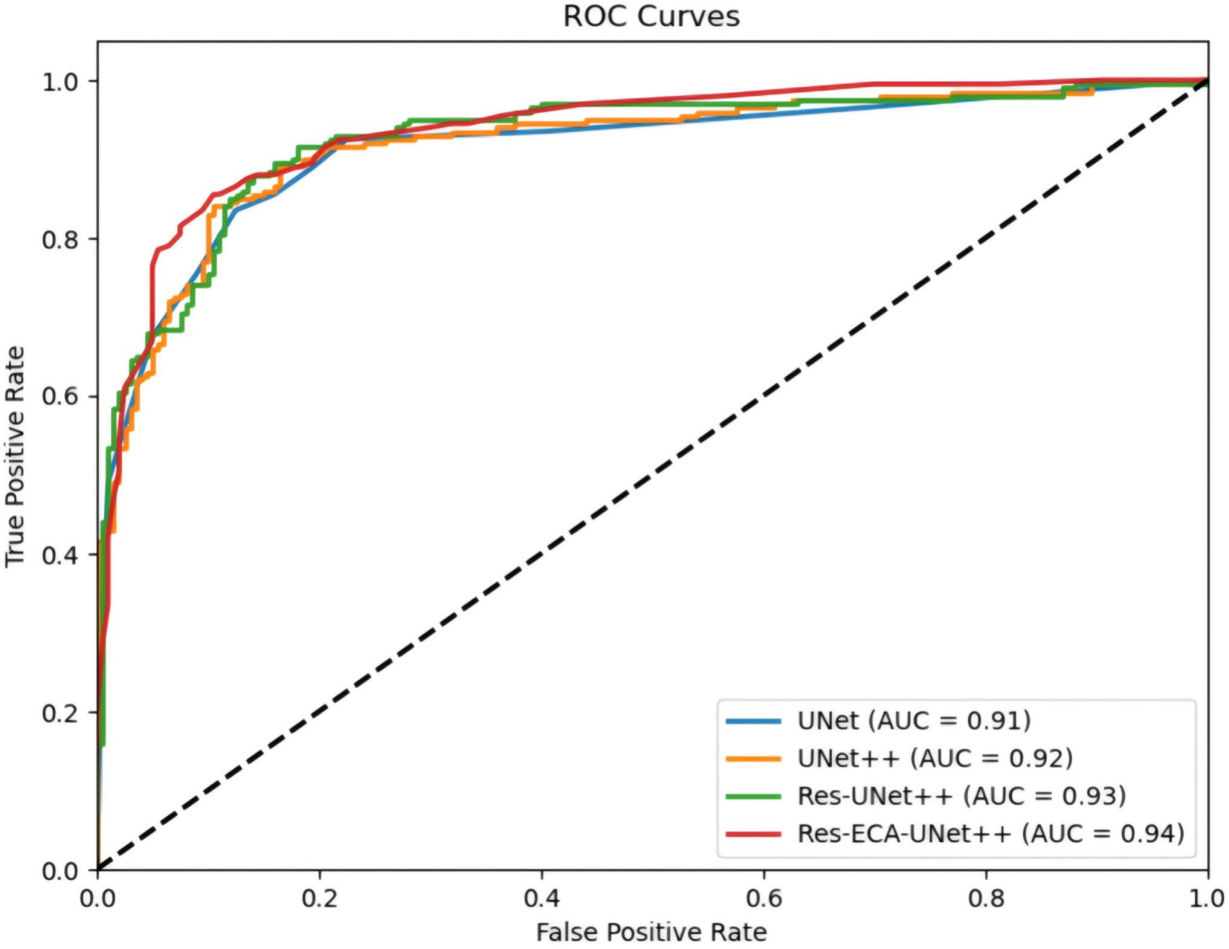
Comparison of ROC curves for different models.

Figure 6 compares the ROC curves of UNet, UNet++, Res-UNet++, and Res-ECA-UNet++ on the test set. The Receiver Operating Characteristic (ROC) curve illustrates the relationship between True Positive Rate and False Positive Rate under varying classification thresholds. Notably, the ROC curve of Res-ECA-UNet++ consistently resides above those of other models, demonstrating its superior performance across different thresholds and faster convergence rate. The Area Under the Curve (AUC) metric, where values approaching 1 indicate better discrimination between positive and negative samples across thresholds, further confirms this advantage. With an AUC value of 0.94, Res-ECA-UNet++ significantly outperforms comparative models, establishing its optimal classification capability.

#### 4.4.2 Computational efficiency and lightweight strategy analysis

To evaluate the deployment potential of Res-ECA-UNet++ in resource-constrained regions, we tested its computational efficiency on both high-end (RTX 3090 GPU) and low-end (NVIDIA Jetson Nano) devices, comparing it with UNet, UNet++, and Res-UNet++. Evaluation metrics included inference time (ms/image), parameter count (millions), and FLOPs (GigaFLOPs), with detailed results presented in Table 3.

**Table 3:** Comparison of computational efficiency.

Model	Inference time (ms/image, RTX 3090)	Inference time (ms/image, Jetson Nano)	Parameters (M)	FLOPs (G)
UNet	20	150	7.8	15.2
UNet++	22	165	9.2	18.5
Res-UNet++	24	180	10.5	20.1
Res-ECA-UNet++	25	190	10.8	21.3

Res-ECA-UNet++ demonstrated an inference time of 25 ms/image on RTX 3090, marginally higher than UNet++'s 22 ms/image. On Jetson Nano, it required 190 ms/image compared to UNet++'s 165 ms/. This performance gap primarily stems from increased model complexity due to the integration of ECA-Net and ResNet architectures, which enhance feature representation and generalization capabilities at the cost of elevated computational demands and parameter count.

Notably, Res-ECA-UNet++ maintains a favorable balance between performance and efficiency, with 10.8 M parameters and 21.3G FLOPs. This configuration preserves high diagnostic accuracy while maintaining practical computational efficiency. The 190 ms/image inference time on Jetson Nano approaches clinical real-time requirements, enabling rapid medical image processing for clinical decision-making. However, further optimizations remain necessary to better adapt to the hardware constraints of portable devices and meet more stringent application scenarios.

To further reduce computational demands, we explored model lightweighting strategies, including network pruning and INT8 quantization. The pruning process, based on L1-norm criteria, removed 30% of low-contribution channels in both ResNet34 and ECA-Net architectures, effectively reducing computational load and storage requirements. INT8 quantization converted the model from FP32 precision to INT8 format, significantly decreasing memory consumption and computational overhead, particularly beneficial for low-end devices. Comprehensive lightweighting experiments were conducted on the test set, with evaluation metrics encompassing Dice coefficient, mIoU, accuracy, Hausdorff distance, inference time, and parameter count, as detailed in Table 4.

**Table 4:** Lightweight experiment results (model: Jetson Nano).

Model Variant	Accuracy (%)	mIoU (%)	Dice (%)	Hausdorff distance (pixels)	Inference time (ms/image)	Parameters (M)	FLOPs (G)
Res-ECA-UNet++ (Original)	99.75	91.84	95.63	7.96	190	10.8	21.3
Res-ECA-UNet++ (Pruned)	99.70	91.24	95.13	8.13	165	7.6	15
Res-ECA-UNet++ (Pruned+INT8)	99.65	90.86	94.80	8.42	140	7.6	14.8

After pruning, the model achieved a parameter reduction to 7.6 M and FLOPs reduction to 15.0G, while maintaining a high Dice coefficient with only 0.53% degradation. The inference time on Jetson Nano accelerated to 165 ms per image. Subsequent INT8 quantization further optimized performance, reducing inference time to 140 ms per image and FLOPs to 14.8G, with the Dice coefficient experiencing a marginal 0.83% decrease and Hausdorff distance slightly increasing to 4.9 pixels - both metrics remaining superior to baseline model performance. Notably, INT8 quantization not only reduced memory requirements but also enhanced inference speed through optimized floating-point to integer conversion. Although these lightweighting strategies incurred acceptable precision losses, they achieved significant computational efficiency improvements. The optimized model demonstrates particular suitability for deployment on resource-constrained devices in low-resource environments, balancing operational efficiency with maintained diagnostic accuracy.

#### 4.4.3 Comparative experiments on improved networks using the OTU2D dataset

To further validate the superiority of the improved network architecture, we conducted comparative experiments on the public OTU2D dataset (46), ensuring both comparability of results and methodological rigor. The experimental outcomes presented in Table 5 reveal that the optimized model achieves superior performance in medical image segmentation tasks, demonstrating strong potential for practical clinical applications.

**Table 5:** Performance comparison of Res-ECA-UNet++ and other models on the OTU2D dataset.

Model	IoU (%)	mIoU (%)	Dice (%)	Hausdorff distance (pixels)	Sensitivity (%)	Specificity (%)
UNet	79.91	86.82	88.91	15.98	87.53	96.81
UNet++	80.53	87.51	89.32	15.13	88.12	97.13
PSPNet (47)	82.01	89.41	90.11	13.98	89.22	97.82
TransUNet (48)	81.31	89.01	90.01	14.42	88.74	97.54
SegFormer (49)	82.46	89.88	90.22	13.52	89.56	98.02
NSBR-Net (33)	82.47	89.89	90.29	13.32	89.81	98.16
Res-ECA-UNet++	82.51	89.91	90.41	13.11	90.21	98.29

#### 4.4.4 Clinical integration and implementation strategies

To optimize the clinical utility of the proposed model, we developed a systematic integration framework aligned with existing clinical workflows. The Res-ECA-UNet++ architecture is embedded within ultrasound imaging systems to enable real-time tumor segmentation, providing clinicians with instantaneous visualization of tumor boundaries and morphological features, thereby reducing diagnostic time while ensuring high precision. For seamless interoperability, the model generates DICOM-compliant outputs and integrates with Picture Archiving and Communication Systems via RESTful APIs, facilitating standardized data storage, cross-device retrieval, and efficient sharing of medical imaging data. Furthermore, the system automates the extraction of tumor biomarkers to generate standardized diagnostic reports that interface directly with Electronic Medical Record platforms, enhancing documentation accuracy and supporting evidence-based therapeutic planning. To ensure clinical adaptability, a web-based interface allows manual refinement of segmentation results, balancing algorithmic efficiency with physician oversight. All components are designed for deployment within conventional clinical infrastructures, prioritizing implementation feasibility, regulatory compliance, and scalability across diverse healthcare settings. This integrated approach addresses critical challenges in workflow optimization while maintaining clinician-centric adaptability.

#### 4.4.5 Error analysis of the model

**Figure 7:**
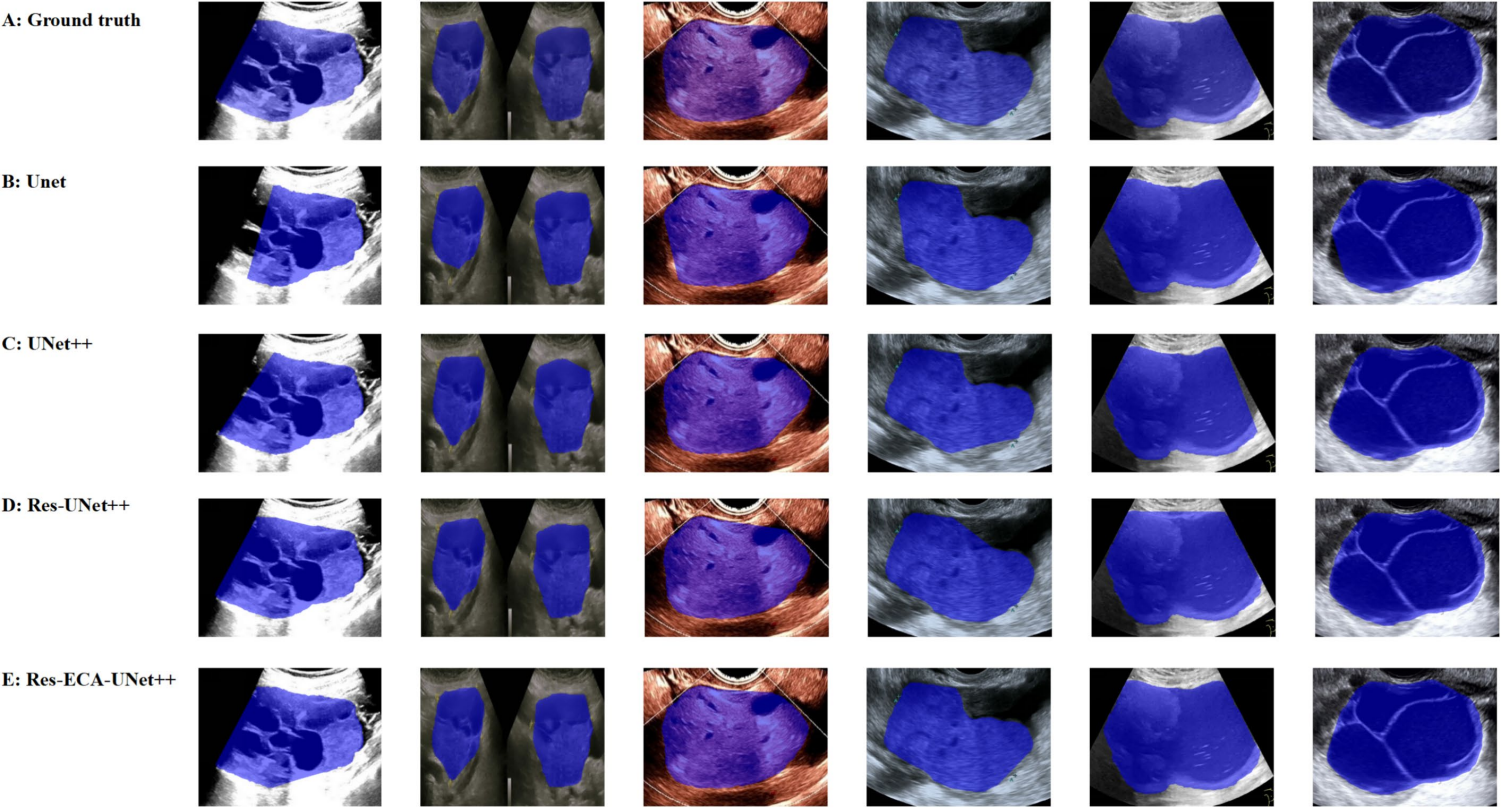
Visual comparison of segmentation results from different models.

While the improved model demonstrates enhanced accuracy in tumor region segmentation, we conducted a visual comparative analysis using representative ovarian cases from our proprietary dataset to further elucidate performance differences across various lesion types. As illustrated in Figure 7, when processing lesions with irregular tumor boundaries, although U-Net series models achieve adequate coverage of major tumor regions, their edge delineation appears coarser with unstable fitting characteristics, particularly showing boundary displacement in low-contrast regions. In comparison, Res-ECA-UNet++ exhibits superior performance in handling most lesion types, though minor boundary deviations persist when processing complex marginal details, indicating potential for improvement in precisely modeling irregular-edged lesions.

Furthermore, the models demonstrate a tendency for over-segmentation when encountering background interference or ambiguous boundary regions, suggesting insufficient structural representation capability at lesion margins. Particularly in areas with regular contours and homogeneous echogenicity, boundary fitting errors reveal the models’ limited robustness in handling transitional boundary ambiguity. Despite Res-ECA-UNet++'s exceptional performance in large lesion identification and boundary precision, optimization potential remains for processing indistinct-bordered lesion types. Future research directions may consider implementing multi-scale modeling mechanisms or boundary enhancement modules to improve the model’s adaptability and robustness when handling highly variable targets.

## 5 Conclusion

With the rapid development of deep learning technologies, U-Net and its derivative models have demonstrated significant clinical value in the field of medical image analysis. This study proposes an improved segmentation model, Res-ECA-UNet++, which addresses gradient vanishing issues and preserves shallow-layer detail features through the integration of residual learning modules and skip connections. The increased network depth enhances both training efficiency and segmentation accuracy. Furthermore, the incorporated ECA-Net channel attention mechanism utilizes 1D convolution to achieve cross-channel interactions, enabling dynamic feature weight adjustment that enhances focus on tumor regions and significantly improves segmentation precision. Experimental results on an ovarian tumor ultrasound dataset demonstrate that Res-ECA-UNet++ surpasses U-Net++ in key metrics including Dice and mIoU, showing notable improvements in segmentation performance. These findings validate its potential for semantic segmentation tasks in ovarian tumor imaging. Regarding computational efficiency, while the model’s increased complexity leads to longer inference times, Res-ECA-UNet++ maintains practical applicability on both high-end and low-end hardware. Through lightweight optimization strategies, the model achieves enhanced inference speed and reduced computational demands while preserving accuracy, demonstrating adaptability for deployment in resource-constrained environments.

While the proposed model demonstrates promising performance, it has several limitations. First, the dataset was collected from a single medical center, which may impact the model’s stability in cross-institutional applications. Second, the current model primarily focuses on common ovarian tumor types and lacks comprehensive validation on rare subtypes, atypical lesions, and complex pathological morphologies—limitations that could affect its generalization in more challenging clinical cases. Additionally, the training protocol relies solely on 2D B-mode grayscale ultrasound images, without sufficiently incorporating structural and functional information from multimodal imaging.

Therefore, future research will focus on the following improvements: (1) Collaborating with multiple medical institutions to collect multicenter data for enhanced generalization; (2) Integrating multimodal ultrasound information and exploring cross-modal transfer learning strategies to improve diagnostic accuracy and robustness in multisource data fusion; (3) Introducing multiscale modeling mechanisms and boundary enhancement modules to refine model robustness, alongside lightweight designs such as network pruning and knowledge distillation for real-time applications, thereby reducing computational demands and facilitating deployment on ultrasound devices.

This research provides an efficient and scalable solution for intelligent ovarian tumor screening. The proposed future enhancements aim to advance the clinical translation and practical adoption of this methodology.

## Data Availability

The raw data supporting the conclusions of this article will be made available by the authors, without undue reservation.
